# Study on the biodegradation of crude oil by free and immobilized bacterial consortium in marine environment

**DOI:** 10.1371/journal.pone.0174445

**Published:** 2017-03-27

**Authors:** Qingguo Chen, Jingjing Li, Mei Liu, Huiling Sun, Mutai Bao

**Affiliations:** 1 College of Marine Science & Technology, Zhejiang Ocean University, Zhoushan, P. R. China; 2 Key Laboratory of Marine Chemistry Theory and Technology, Ministry of Education, Ocean University of China, Qingdao, P. R. China; MJP Rohilkhand University, INDIA

## Abstract

Five strains of bacteria, namely, *Exiguobacterium* sp. ASW-1, *Pseudomonas aeruginosa* strain ASW-2, *Alcaligenes* sp. ASW-3, *Alcaligenes* sp. ASS-1, and *Bacillus* sp. ASS-2, were isolated from the Zhejiang coast in China. The mixed flora of the five strains performed well with degrading 75.1% crude oil (1%, *w/v*) in 7 days. The calcium alginate—activated carbon embedding carrier was used to immobilize bacterial consortium. Immobilized cells performed better than free ones in variations of environmental factors containing incubated temperature, initial pH, salinity of the medium and crude oil concentration. The degradation process of crude oil by immobilized bacteria was accelerated compared with that of the free ones. Bacterial consortium showed better performance on biodegradation of normal alkanes than that of PAHs. Improvement of immobilization on the biodegradation efficiency of normal alkanes (31.9%) was apparently high than that of PAHs (1.9%).

## Introduction

Recently, with the development of offshore oil and transportation industry, the probability of spill oil is increasing on offshore oil platforms, oil tanker, and offshore oil facilities. Many oil drilling platforms are located in China's offshore shipping and port areas, and they are not only the national strategic oil reserve base but also the oil reserve base of Chinese oil companies. Thus, the problem of oil leakage and pollution in coastal waters and ports has persisted for a long time. [1]

Bioremediation technology was used to degrade marine oil spill during the 1970s. [2] The technology was considered as the best way of restoring the ecological environment of the sea due to its low cost, environmental friendliness, and absence of secondary pollution. [3, 4] However, problems have been encountered in the field of application of bioremediation technology due to uncontrollable factors, such as the loss of effective strains and substantial decline in the efficiency of remediation, because the ocean is an open and highly mobile system. Immobilized microbial technology is favored for its high cell concentration, low microbial loss, enhanced tolerance of the environment, and high degradation efficiency of petroleum hydrocarbons. [5] This way, the difficulties brought about by the unique environment of the ocean can be overcome. Therefore, the bacterial consortium in this study was immobilized to study the degradation efficiency of crude oil by free and immobilized bacteria.

Embedded immobilization technique, as a method of immobilization, is widely used for its simple operation, mild and easy to be realized reaction condition, low probability of microorganism leakage, good performance at stability and reuse, high microbial activity and cell capacity, and high immobilization efficiency which can reach more than 70% compared with other immobilization technologies, such as adsorption, cross-linking, and covalent bonding. [6, 7] In view of these properties, researchers used embedded immobilization technique to immobilize the microorganisms. [8–11] Sodium alginate-calcium chloride (calcium alginate) embedding and polyvinyl alcohol—boric acid embedding are the most commonly used methods of embedding immobilization. [9] Compared with the calcium alginate embedding method, the immobilized preparation by polyvinyl alcohol-boric acid has a much higher mechanical strength and much longer service life. However, the polyvinyl alcohol—boric acid embedding reduces the activity of microorganisms after significant immobilization due to difficulty in forming the ball caused by the method’s strong agglomeration effects and toxicity of saturated boric acid solution of polyvinyl alcohol used in cross-linking. [10] Although the embedding method of calcium alginate is superior in terms of mild reaction conditions, absence of toxicity to the organisms of the embedded materials, and higher activity of encapsulated cells, the mechanical strength of the preparation is low, and the mass transfer performance is poor. [12]

In this study, the performance of preparation calcium alginate bio-carrier was improved by adding activated carbon in the embedding to overcome the low mechanical strength, the lack of mass transfer, [13] and to greatly improve mass transfer because the activated carbon is a porous structure. The objectives of this research are: 1) to start up a lab-scale effective petroleum hydrocarbon degrading microorganisms from oil-contaminated seawater samples for marine oil pollution treatment; 2) to explore the characteristics of the immobilized microorganisms; 3) to investigate the performance of immobilized microorganisms on biodegradation of normal alkanes (*n*-alkanes) and polycyclic aromatic hydrocarbons (PAHs) in marine environment.

## Materials and methods

### Materials

#### Chemical reagents

The standard chemicals, including naphthalene, acenaphthylene, acenaphthene, fluorene, phenanthrene, anthracene, fluoranthene, pyrene, benzo(a)anthracene, chrysene, benzo(b)fluoranthene, benzo(k)fluoranthene, benzo(a)pyrene, indeno(1,2, 3-cd)pyren, dibenzo(a,h)anthracene, benzo(g,h,i)perylene, and their deuterated products, and normal alkanes mixed labeled as C8–C33 and C_24_D_50_, were obtained from Sigma-Aldrich Co LTD, USA. The rest of the chemical reagents used in this research were obtained from China National Pharmaceutical Group.

The crude oil sample was from Xingzhong sinochem oil transport (Zhoushan) Co., Ltd, China. Its density is about 0.91 g·cm^-3^.

The crude oil used in the experiment was obtained by dissolving crude oil sample in non-aromatic petroleum ether (60–90°C), and then desalinated using anhydrous sodium sulfate, and finally filtrated. The filtrate was vaporized at (45±1°C on water bath.

#### Media

1. Seawater medium (SM). 0.2 g of NH_4_Cl, and 0.2 g of K_2_HPO_4_ were added into 1000 mL of seawater with a pH adjusted to 8.0.
2. Artificial seawater medium (ASM). 19 g of NaCl, 7.0 g of MgSO_4_·7H_2_O, 1.0 g of NH_4_NO_3_, 2.0 g of KH_2_PO_4_, 3.0 g of Na_2_HPO_4_, and 0.7 g of KCl were added into 1000 mL of deionized water, with a pH adjusted to 8.0. [14] Trace elements were added into the medium with 10 mL per litre. The trace elements contained (in grams per litre) the following compounds 0.06 MnSO_4_·H_2_O, 0.5 FeSO_4_·7H_2_O, 0.2 ZnSO_4_·7H_2_O.
3. Crude oil medium. 1–10% (*w/v*) of crude oil with SM or ASM.
4. Broth medium. 10 g of peptone, 3g of beef extract added into 1000 mL of seawater, with a pH adjusted to 8.0.
5. Broth agar medium. 25 g of agar were added into 1000 mL of broth medium.

The seawater used in this experiment was sampling from sea area (29.96° N, 122.19° E) near Changzhi Island, Zhejiang province. The concentration of petroleum hydrocarbons in the seawater was lower than 0.02 mg/L. It was subjected to boiling at 100°C for 30 min and sucking filtration by glass fiber filter with pore size of 0.45μm before being used in media preparation. All the media were sterilized at 121°C for 20 min before use.

#### Seawater sample collection for screening of petroleum hydrocarbon degrading bacteria

The crude oil polluted seawater was collected from an area near an oil terminal in Zhejiang province, the geographic coordinates for the sampling location is 30.61° N, 122.14° E. The collected samples were placed in sterilized glass bottles and stored at 4°C after they were labeled.

We just only sampled seawater for scientific research from the sampling locations mentioned above, and no specific permits were required for the described field studies in China. The locations were not privately owned or protected, and the field studies did not involve endangered or protected species.

### Screening and isolation of petroleum hydrocarbon degrading bacteria

#### Separation of petroleum hydrocarbon degrading microorganisms

The separation of petroleum hydrocarbon degrading bacteria was as follows. 100 mL of crude oil medium (SM) and 0.2 g/mL yeast extract powder were placed in a clean conical flask, and 2 mL of the collected oil polluted seawater samples was injected by sterile syringe to the crude oil medium on the clean workbench which was sterilized. The medium was cultured for 3 d in the constant temperature shaker at parameters 150 r/min and 25°C. Then, 2 mL of cultured medium was transferred to the newly sterilized 100 mL of crude oil medium and cultured under the same conditions for 3 d. The liquid was coated in broth agar medium after repeating the aforementioned operation twice. The eruptive colony was picked up by inoculation loop after it has been spread plate cultivated. The colony which grew fast and well were lined separately on a flat plate and finally cultured at 25°C for 1–2 d. Then, the above-mentioned steps were repeated until a single pure bacterial appearance was observed. The purified strains were preserved in broth agar slant culture at 4°C, and ready for use.

The preliminary morphological observation and physiological and biochemical tests on screening bacterial strains were carried out according to procedures as described by Shen and Chen (2013). [15]

#### Strain culture and sequence

The screening petroleum degrading bacteria were inoculated with 2 mL/100 mL ratio in liquid crude oil medium and then incubated in constant temperature oscillation incubator for 7 d in the constant temperature shaker at parameters 150 r/min and 25°C.

After 24 h, the purified strains cultured in enriched medium were preserved via cryogenic refrigeration at 4°C, and the cells were harvested by centrifuging the enriched culture at 9750×g. The pellet was then washed with phosphate buffer solution and centrifuged thrice after the medium was removed and used for DNA extraction and purification. The amplified sequencing of 16S rDNA was completed by PCR (Bio-Rad, USA) using the obtained DNA.

PCR amplification was performed in a 25 μL reaction mixture containing 2.5 μL of 10× PCR buffer, 0.5 μL of primers 27f, 0.5 μL of primers 1492R, 0.2mM concentration for deoxy-nucleotide triphosphates (dNTP), 0.2 μL of Taq DNA polymerase (5U) and template DNA (1 μL). All the reagents were supplied by Nanjing Genscript Biotechnology Co., Ltd, China. PCR was performed under the following conditions: initial denaturation at 94°C for 5 min, 30 cycles of 94°C for 30 s, 61–65°C for 30 s, and 72°C for 60 s in a MyCycler Thermal Cycler (BIO-RAD) and a final extension at 72°C for 10 min. Amplicons were detected by electrophoresis on a 1% agarose gel staining with ethidium bromide. Amplicons were purified using a TIANamp Bacteria DNA kit eluted in Tris—HCl (10 mM, pH 8.5) prior to sequencing. The sequencing was completed by Nanjing Genscript Biotechnology Co., Ltd, China.

The sequence obtained was identified using the Basic Local Alignment Search Tool of the GenBank of National Center for Biotechnology Information, and phylogenetic trees were constructed based on the neighbor-joining method by bootstrap test using MEGA 4.0 software.

### Crude oil degradation efficiency

Petroleum degradation efficiency was determined by spectrophotometric method described as Bao et al. (2013). [14] The non-aromatic petroleum ether (60–90°C) was used for sample extraction. And it was used as reference at the same time.

### Bacterial suspension preparation

The bacteria after incubated in broth medium at 25°C and 150 r/min for 24 h were centrifuged at 9750×g centrifugal force to acquire the bacterial cells, these cells were washed by seawater and centrifuged for 3 times, in order to remove the nutrition in the culture broth. These purified cells mixed with seawater to get the bacterial suspension, which was 1 of absorbance adjusted via absorbance at 600 nm (the biomass was approximately of 3×10^10^ cell/mL), and then the bacterial suspension was ready for use. The prepared bacterial suspension was used as inoculation fluid for all the crude oil biodegradation experiments.

### Construction of bacterial consortium

The oil degradation experiment was done with the five strains and combination among them. The initial inoculation quantity was 5 mL bacterial suspension of 45 mL crude oil medium (SM) of each set according to the principle of equal quantity. The crude oil concentration was 1% (*w/v*). The oil degradation efficiency was determined after 7 d’s shake cultivation. The bacterial consortium which has the highest crude oil degradation efficiency was considered the optimal bacterial consortium used in the following experiment.

### Immobilization of free microorganisms

#### Method of preparing calcium alginate—Activated carbon microsphere

1. Sodium alginate, activated carbon, and calcium chloride were taken into a clean working platform after sterilization at 121°C during 15 min;
2. 0.75 g activated carbon were added into 50 mL bacterial suspension prepared as described in Section 2.5 and were mixed for 15 min. Then, 2.0 g sodium alginate was added into the mixture and was mixed for 15 min again;
3. The mixture in Step 2 prepared was extruded through a peristaltic pump to calcium chloride solution (3%, *w/v*) for cross linking for 12 hours to form spherical beads; and
4. The immobilized microsphere was rinsed with sterile seawater and then preserved in sterile seawater at 4°C until further use. [16]

#### Scanning Electron Microscopic (SEM) examination of immobilized cells

The bio-carriers and immobilized cells were firstly fixed with 2.5% glutaraldehyde for 2 h, in order to carry out SEM observation. Then, they were dehydrated for 30 min by a series of gradually increasing ethanol concentrations: 10, 30, 50, 70, 80, 90, and 100%, respectively. The bio-carriers were dried using the CO_2_ critical point drying technique and then were coated with gold. [17] The morphology of immobilized bacterial cells were observed using scanning electron microscopy (KYKY-2800B, KYKY Technology CO., LTD., China) according to a standard procedure with an accelerating voltage of 15 kV. [6]

### Experiments of environmental factors of biodegradation

#### Influence of crude oil concentration

45 mL of crude oil medium (SM) was poured into 100 mL triangular flask, and 0.1, 0.2, 0.3, 0.4, 0.5, 0.75, and 1 g of crude oil was added, respectively. Afterward, free bacterial consortium (5 mL bacterial suspension) and immobilized bacterial consortium (5 mL immobilized microsphere) were added into the crude oil medium, respectively, and the concentration of crude oil was determined after 7 d of culturing at 20°C.

#### Influence of liquid temperature

As optimized oil concentration was added in the crude oil medium (SM) described in Section 2.7.1, the incubation temperatures were set at 5, 10, 15, 20, 25, and 30°C, respectively, and the concentration of crude oil was determined after 7 d of culturing.

#### Influence of initial pH

The pH of crude oil medium (SM) was adjusted to 6, 6.5, 7, 7.5, 8, 8.5, and 9, respectively, using 1 mol/L of HCl and NaOH solution. Free and immobilized consortium was inoculated into the crude oil medium, respectively, and the concentration of crude oil was determined after 7 d of culturing at 20°C.

#### Influence of salinity

Crude oil medium (ASM) was used in this experiment. The salinity was regulated to 15, 20, 25, 30, and 35‰ by adjusting the NaCl content. The free and immobilized consortium was inoculated into the crude oil medium, and the concentration of crude oil was determined after 7 d of culturing at 20°C.

#### Influence of degradation time

The free and immobilized consortium was inoculated into crude oil medium (SM) and cultured for 1, 3, 5, 7, and 9 d, respectively, and the concentration of crude oil was determined afterward.

All the biodegradation experiments were carried out in triplicate with the live bacterial cultures and the controls. The oil removal efficiency was acquired as the average value.

### Analysis of crude oil degradation by GC-MS

#### Column chromatography elution of crude oil fractions

Column chromatography was used to separate the main hydrocarbon components from crude oil samples after biodegradation and the control. [18, 19] The *n*-alkanes and PAHs were determined according to the following methods after elution.

#### Determination of n-alkanes

The 5 MS hp-quartz capillary column chromatography was used under the same condition. The normal paraffin hydrocarbons were labeled as C8–C33 (containing Pr and Ph), with the C_24_D_50_ used as internal standard. The quantitative ion peak of normal alkanes is 171, and the qualitative ion peaks are 71.1, 85.1, and 99.1. The quantitative ion peak of C_24_D_50_ is 66.1, and the qualitative ion peak is 82.1 and 98.1.

#### Determination of PAHs

The sample was analyzed by gas-chromatography (GC-FID) under the following conditions. The Agilent 6890N gas-chromatograph was fitted with a splitless injector, a fused-silica capillary column (HP-5MS, 30 m × 0.25 mm × 0.25 μm). The condition of the chromatography used split/non shunt inlet at 280°C. The sample was placed in the spiritless injector for 1 min and was heated with 1.0 mL/min of high purity helium gas and constant current. After that the temperature was set at 70°C and kept for 2 min, and then the temperature was raised to 140°C at 25°C/min. The temperature was further increased to 240°C at 3°C/min until the temperature reached 300°C at a rate of 10°C/min. The sample was retained for 5 min. The transmission line temperature was 280°C.

The conditions for mass spectrometry analysis were as follows. Positive ion mode was chosen as EI source, and the temperature of ion source is 230°C. The analysis was conducted at 50 μA of alternating current, 1.5 millitorr of impact gas (argon) pressure, and by using SRM scan pattern. The PAHs used for qualitative and quantitative measurements were selected based on those which are commonly found in marine environments: naphthalene, acenaphthylene, acenaphthene, fluorene, phenanthrene, anthracene, fluoranthene, pyrene, benzo(a)anthracene, chrysene, benzo(b)fluoranthene, benzo(k)fluoranthene, benzo(a)pyrene, indeno(1,2,3-cd)pyren, dibenzo(a,h)anthracene, and benzo(g,h,i)perylene. These 16 kinds of PAHs are listed as environmental priority persistent organic pollutants by the United States Environmental Protection Agency in the 1980s. [20] China has also been included in the blacklist of producer of environmental pollutants.

Therefore, we focus on the microbial degradation of these 16 kinds of PAHs in crude oil. The charge mass ratio for the qualitative and quantitative ion peaks and the quality control of chromatographic conditions were observed according to the study of Chen et al. (2013). [21]

## Results

### Isolation and identification

Five oil-degrading microorganisms ASW-1, ASW-2, ASW-3, ASS-1 and ASS-2 were isolated from crude oil polluted seawater samples. The experiment proved that all the five strains were capable to grow in crude oil by using petroleum hydrocarbon as the sole carbon source.

Table 1 presented the results of the physiological and biochemical tests used to characterize five microorganisms. They were identified taxonomically further by 16S rDNA gene amplification and sequencing. Approximately 1500 bp sized-fragment of the 16S rDNA gene of each isolate was amplified and sequenced. Sequence analysis of the 16S rDNA gene and BLAST sequence comparison confirmed that the isolated strains ASW-1, ASW-2, ASW-3, ASS-1 and ASS-2 were affiliated with *Exiguobacterium sp*. *Pseudomonas aeruginosa*, *Alcaligenes sp*., *Alcaligenes sp*., and *Bacillus* sp., respectively. The nucleotide sequences of 16S rDNA of ASW-1, ASW-2, ASW-3, ASS-1 and ASS-2 determined in this study had been deposited in the GenBank database under accession numbers KM243657, KM243658, KM243659, KM243661 and KM243662, respectively.

**Table 1:** Morphology and biochemical characterization of the strains.

strain	1#	2#	3#	4#	5#
colony morphology	yellow	canary yellow	white translucent	white translucent	white
colony size	small	small	small	small	big
Surface wetting	+	+	+	+	-
gram stain	-	-	-	-	+
flagella stain	+	+	+	+	+
spore stain	-	-	-	-	+
starch hydrolysis test	+	-	-	-	-
Sugar fermentation test	-	-	+	-	+
methyl red test	+	+	+	-	+
V-P test	+	-	-	-	-
indole test	-	+	+	+	-
catalase test	+	+	+	+	+

+, positive; −, negative.

**Fig 1:**
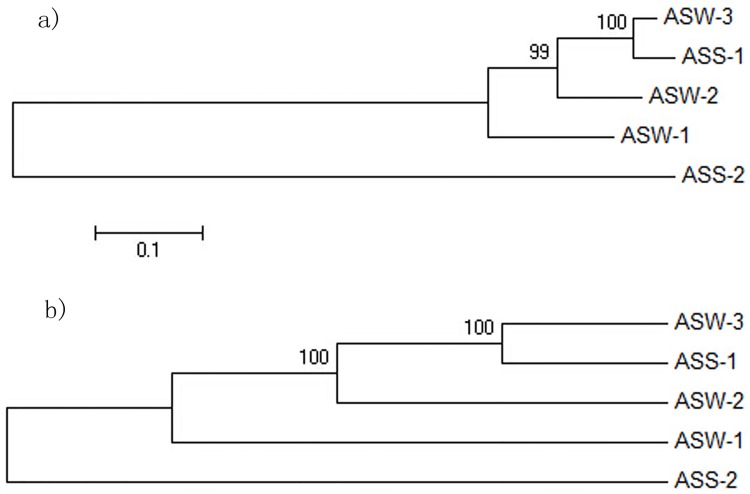
Phylogenetic tree of the strains based on bootstrap test. a) A neighbor-joining method; b) A maximum parsimony method.

In order to carry out the homology analysis of the five strains of bacteria, we used the neighbor-joining method and MP (bootstrap test) to construct a phylogenetic tree. Results are shown in Fig 1. From the constructed phylogenetic trees, we can conclude that ASS-1, ASW-1, ASW-2, and ASW-3 are closely related, and ASS-1 and ASW-3 showed the closest relationship, whereas ASS-2 and the other four strains were the least related.

### Construction of optimal mixed flora

The bacterial consortium constructing was done based on the five strains in order to get better degrading performance of crude oil. All the combinations of the five strains and their degradation abilities show in Table 2.

**Table 2:** Degradation efficiencies of bacterial consortia and single strains.

order	groups	inoculum volume(ml)					degradation efficiency(%)
		1#	2#	3#	4#	5#	
1	individual strain	5	0	0	0	0	50.5±2.1%
2		0	5	0	0	0	65.4±1.3%
3		0	0	5	0	0	60.2±0.5%
4		0	0	0	5	0	55.5±1.5%
5		0	0	0	0	5	51.4±0.8%
6	two strains	2.5	2.5	0	0	0	53.3±3.0%
7		2.5	0	2.5	0	0	48.7±2.3%
8		2.5	0	0	2.5	0	67.2±4.1%
9		2.5	0	0	0	2.5	58.5±1.4%
10		0	2.5	2.5	0	0	70.4±1.1%
11		0	2.5	0	2.5	0	56.6±2.3%
12		0	2.5	0	0	2.5	51.4±1.6%
13		0	0	2.5	2.5	0	71.8±2.2%
14		0	0	2.5	0	2.5	60.5±1.5%
15		0	0	0	2.5	2.5	67.2±0.6%
16	three strains	1.67	1.67	1.67	0	0	62.6±1.9%
17		1.67	1.67	0	1.67	0	70.8±1.3%
18		1.67	1.67	0	0	1.67	41.6±0.9%
19		0	1.67	1.67	1.67	0	70.1±2.6%
20		0	1.67	1.67	0	1.67	44.2±3.2%
21		0	0	1.67	1.67	1.67	41.9±1.7%
22		0	1.67	0	1.67	1.67	54.2±3.5%
23		1.67	0	0	1.67	1.67	72.2±2.7%
24		1.67	0	1.67	0	1.67	55.4±1.5%
25		1.67	0	1.67	1.67	0	58.4±2.6%
26	four strains	1.25	1.25	1.25	1.25	0	71.9±3.4%
27		1.25	1.25	1.25	0	1.25	64.2±4.7%
28		1.25	1.25	0	1.25	1.25	71.9±1.4%
29		1.25	0	1.25	1.25	1.25	69.7±2.3%
30		0	1.25	1.25	1.25	1.25	57.4±1.2%
31	five strains	1	1	1	1	1	75.1±1.9%

From Table 2, the degradation efficiencies of the five pure strains in the seawater medium containing 1% (*w/v*) crude oil were 50.5–65.4%. The combinations of two, three and four strains got the degradation efficiency of 41.6–72.2%, and the mixed flora of the five strains showed the highest degradation efficiency of petroleum hydrocarbons of 75.1%. The optimal mixed bacterial consortium was used in the following research.

### SEM of bio-carrier and immobilized bacterial consortium

**Fig 2:**
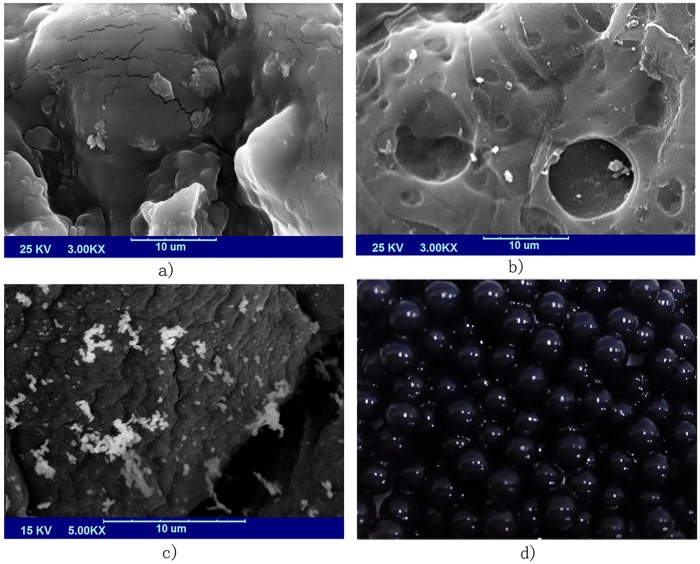
Photos of bio-carrier and immobilized bacterial consortium. a) SEM photo of bio-carrier of calcium alginate; b) SEM photo of bio-carrier of calcium alginate—activated carbon; c) SEM photo of immobilized bacterial consortium; d) Photo of immobilized bacterial consortium microsphere.

SEM photos of bio-carrier of calcium alginate, bio-carrier of calcium alginate—activated carbon and immobilized bacterial consortium are shown in Fig 2. Compared with bio-carrier of sodium alginate-calcium chloride (Fig 2a), activated carbons adding in the bio-carrier (Fig 2b) not only made the carrier scabrous on interior surface, but also brought around multiple porous structures that profited the diffusion of oxygen, substrates and metabolites. [6] Fig 2c showed bio-carrier of immobilized bacterial cells. It could be seen the bacterial consortium has been adhered to the interior surface of the bio-carrier and ready for biodegradation. The photo of Fig 2d was the appearance of prepared immobilized bacterial consortium microspheres. It was about 3–4 mm in diameter. Due to the larger specific surface area and higher adsorption capacity of activated carbon, the bacterial colonization and in-suit bioremediation were promote by supplying an available surface area to support bacterial growth and adsorbing of sufficient substrates to make a direct contact between bacterial cells and petroleum hydrocarbon to make a better performance in crude oil biodegradation. [17]

The crude oil adsorption capacity of bio-carrier was determined. 86.1% of crude oil removal efficiency was obtained by the immobilized cells incubated in crude oil medium for 7d with 1% (*w/v*) crude oil and 5% (v/v) inoculum dose, while only about 5.7±1.2% was adsorbed by the bio-carrier. Biodegradation of bacterial consortium in bio-carrier was the main moving force to crude oil removal, and the bio-carrier provided an arena for bacterial cells protection and better biodegradation. The influence of environmental factors on free and immobilized bacterial consortium biodegradation was conducted in the following study.

### Effect of environmental factors on crude oil biodegradation

#### Crude oil concentration

In the bioremediation process, the concentration of pollutants affects microbial degradation, and too high or too low concentration leads to the decrease in the efficiency of microbial degradation.

**Fig 3:**
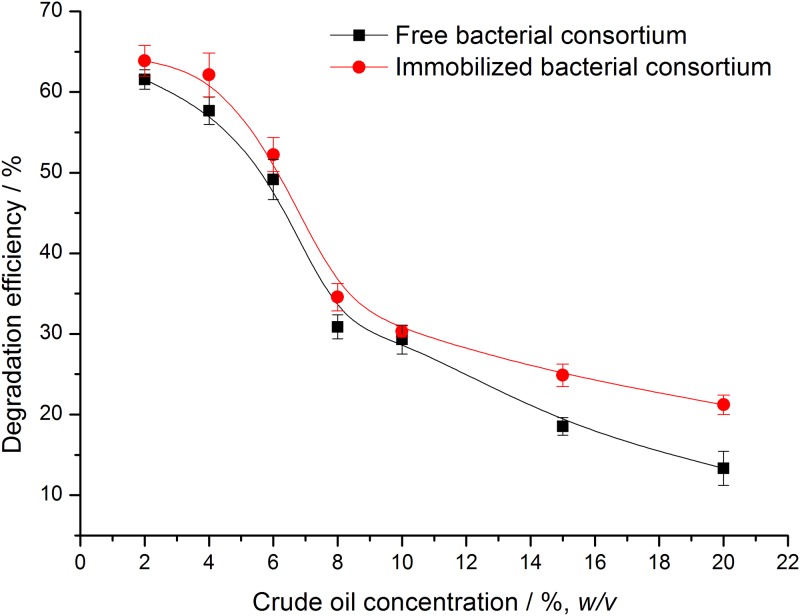
Effects of crude oil concentration on crude oil degradation efficiency of free and immobilized bacterial consortium.

As seen in Fig 3, the crude oil concentration greatly influenced the degradation of crude oil by the free and the immobilized bacterial consortium. The increase in the concentration of the crude oil led to the decrease in the degradation by free and immobilized bacterial consortium. However crude oil degradation decreased when concentrations of crude oil were increased, [22] the degradation efficiencies of immobilized bacterial consortium were still high than those of the free bacterial consortium. The degradation efficiency of the free and immobilized bacterial consortium reached maximum values of 58.6% and 63.9%, respectively, at the oil concentration of 2% (*w/v*). When concentration of crude oil increased from 2% to 4% (*w/v*), the degradation efficiency of immobilized bacteria only decreased by 1.76%, compared with 3.9% by free bacteria. The degradation efficiency of immobilized bacteria also began to significantly reduce followed by the concentration of crude oil increased from 4% to 10% (*w/v*). Although the high concentration of crude oil inhibit the activity of microorganism, the degradation ability of immobilized increase significantly relative to that of free bacteria, followed by crude oil concentration more than 10% (*w/v*). When the concentration of crude oil was 20% (*w/v*), the degradation efficiency of immobilized bacteria increased by 7.9% compared with that of the free bacteria. The crude oil content was chosen for 2% (*w/v*) in the following studies. The biodegrability of free and immobilized cells were explored under different incubated temperature, initial pH, and salinity.

#### Temperature

Temperature not only affects the activity of bacteria, it also influences the physical and chemical properties of crude oil in the ocean and the degradation ability of bacteria. The change in the temperature of Yangtze Estuary and adjacent areas of China is generally between 8–30°C, [23] and the area in which the average annual water temperature over 20°C. We investigated the influence of degradation efficiency of free and immobilized bacterial consortium at 5–30°C.

**Fig 4:**
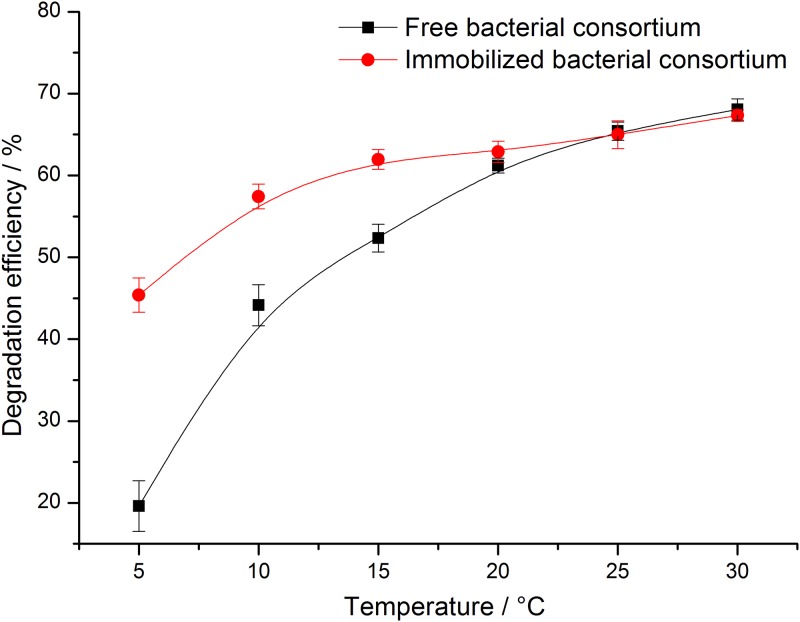
Effects of temperature on crude oil degradation efficiency of free and immobilized bacterial consortium.

As shown in Fig 4, the degradation ability of free and immobilized bacterial consortium all increased with increasing temperature. When the temperature was 30°C, the efficiencies reached the maximum, and their values were 68.1% and 67.3%, respectively. When the temperature was 5°C, the degradation efficiencies of the free and immobilized bacterial consortium were both minimum and the efficiencies were 19.6% and 45.4%, respectively. The minimum degradation efficiency was greatly improved after immobilization, and the efficiency increased by 25.8%, which can be used in situation when low temperature is not conducive to microbial degradation.

#### Initial pH

pH is one of the important environmental factors affecting the growth of bacteria. The growth of bacteria is seriously affected in the presence of unsuitable environmental pH environment. Moreover, the ability of the degradation of bacteria is also affected. [24]

**Fig 5:**
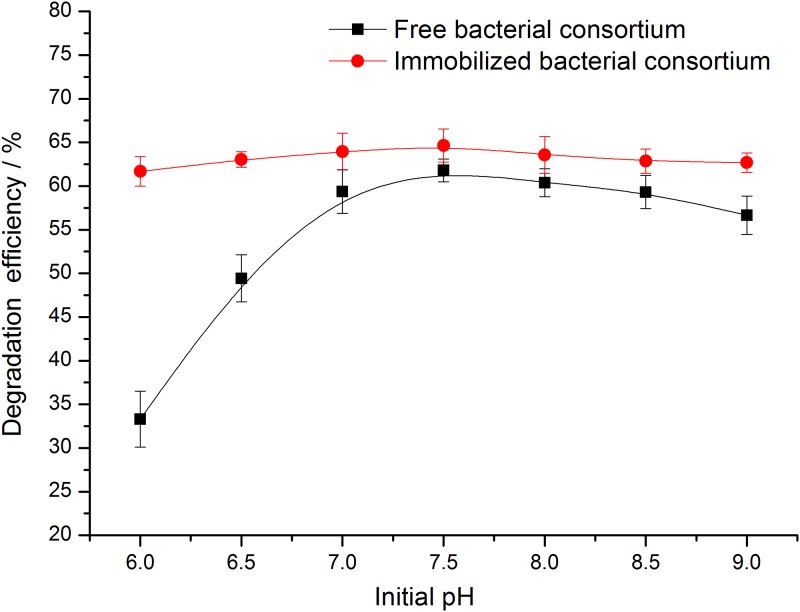
Effects of initial pH on crude oil degradation efficiency of free and immobilized bacterial consortium.

As seen in Fig 5, the degradation efficiency of crude oil of free and immobilized bacterial consortium increased initially and decreased with increasing initial pH of the medium. When the initial pH was 7.5, the degradation efficiency of the free bacterial and the immobilized bacterial consortium reached the maximum values of 61.8% and 64.6%, respectively. The degradation efficiency of the free bacteria consortium was affected apparently by the initial pH, and the variation range of the degradation efficiency is up to 28.5%. However, after immobilization, the variation range of degradation efficiencies was less than 4.3% with initial pH of the medium changed from 6 to 9. Immobilization not only improves the biodegradation efficiency of crude oil, it also expands the range of pH suitable for microorganisms. [25]

#### Salinity

**Fig 6:**
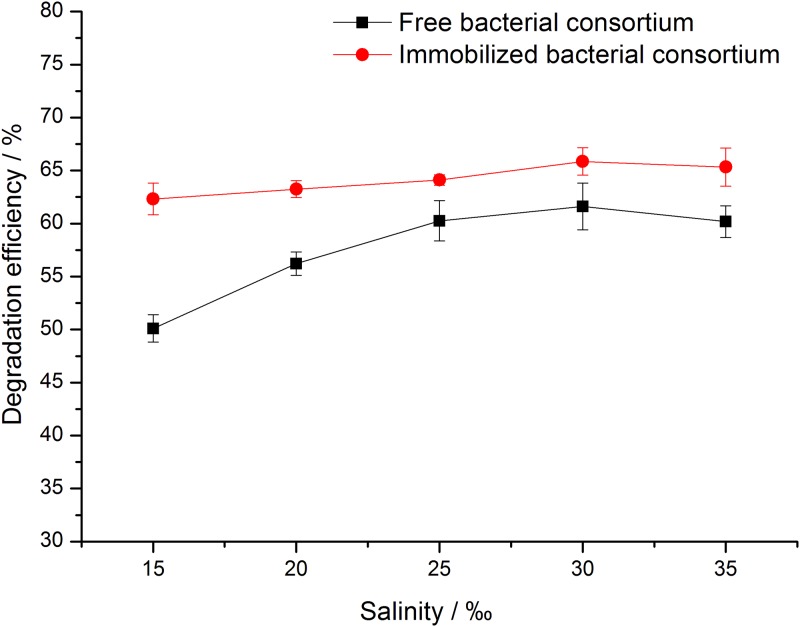
Effects of salinity on crude oil degradation efficiency of free and immobilized bacterial consortium.

To confirm that immobilized cells can also tolerate a wider salinity changes, crude oil biodegradation experiments was performance at a salinity range from 15 to 35‰, and the results are shown in Fig 6. The effect of salinity on the degradation of crude oil by free microorganisms was affected apparently. The degradation efficiency of free bacterial consortium increased initially and then decreased with increasing salinity. The optical salinity was 30‰ by naturally existing bacteria isolated from the marine environment. A similar study has been reported by Pasumarthi et al. (2013). [26] When the salinity was 30‰, the degradation efficiency of crude oil of free bacterial consortium reached the maximum values of 61.6%. After immobilization, the degradation efficiencies were improved by 3.9–12.2%. The variation range of degradation efficiencies of immobilized cells was less than 3.1% with salinity of the medium changed from 15 to 35‰, while that of free cells was more than 10%. The bacterial consortium not only increases the degradation efficiency of crude oil but also enhances tolerance toward salinity of the bacteria. [13]

#### Degradation time

**Fig 7:**
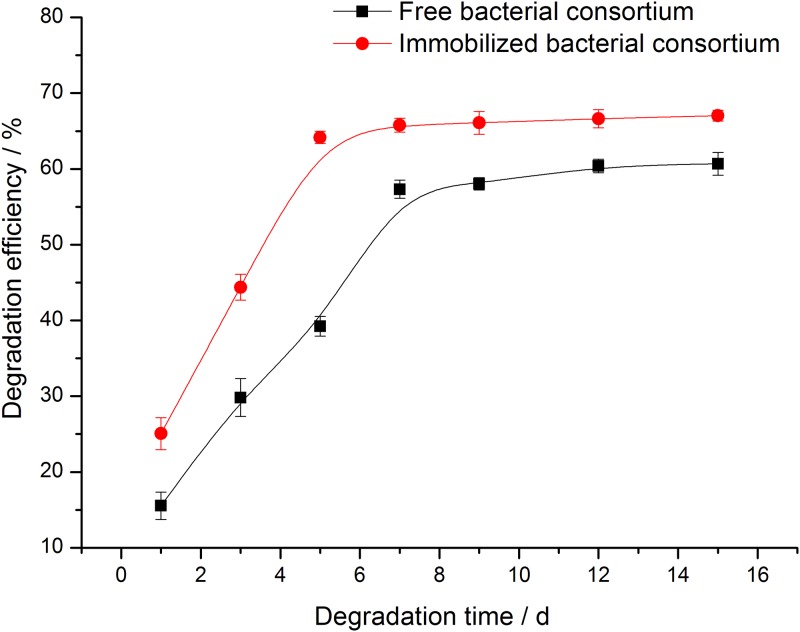
Effects of degradation time on crude oil degradation efficiency of free and immobilized bacterial consortium.

Fig 7 shows the change in the degradation efficiency of crude oil with time by free and immobilized bacterial consortium. The degradation efficiency of crude oil increased firstly and then stabilized with time [6, 27]. The degradation of crude oil by free bacterial consortium was mainly concentrated in the first 7 d with degradation efficiency of 57.3%, and that of the immobilized bacterial consortium was mainly concentrated during the first 5 d with degradation efficiency of 65.9%. The degradation process of crude oil by immobilized bacteria was accelerated compared with that of the free bacteria.

### Analysis of crude oil biodegradation by GC-MS

#### Normal alkanes

**Fig 8:**
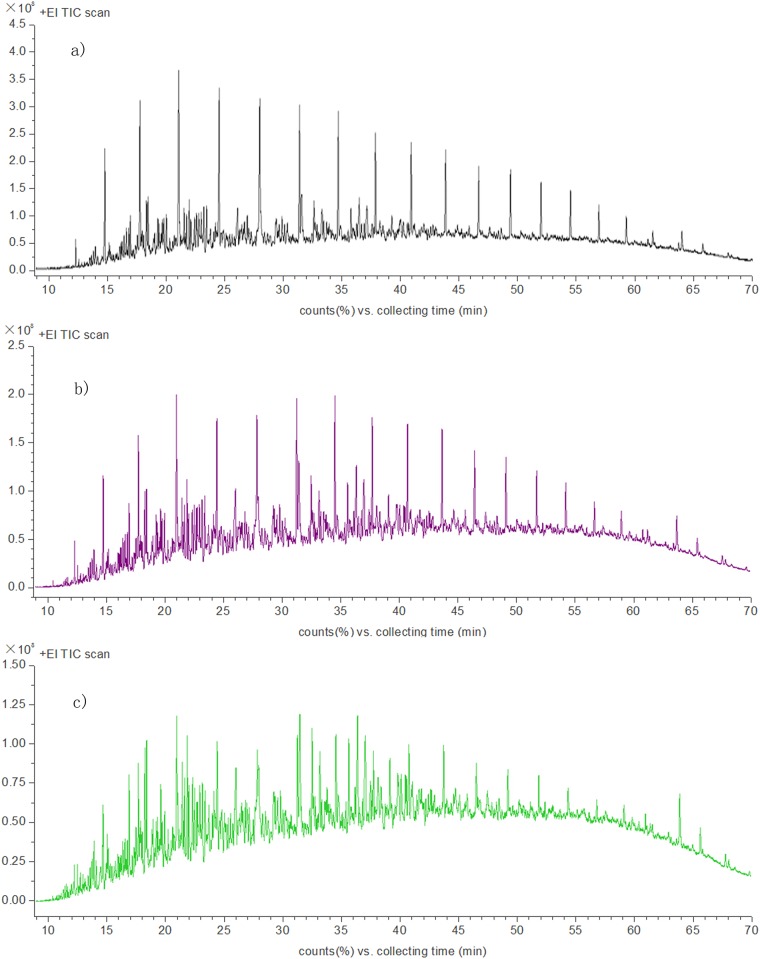
n-alkanes gas chromatograms in crude oil samples before and after biodegradation. a) the crude oil control; b) degradation sample of free bacterial consortium; c) degradation sample of immobilized bacterial consortium.

**Fig 9:**
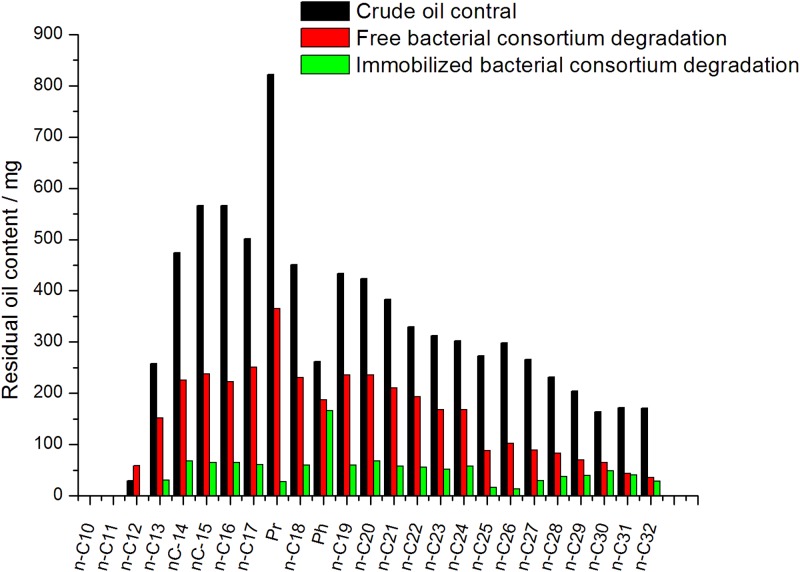
Changes of the major normal alkanes content in crude oil samples before and after biodegradation.

Gas chromatograms of the crude oil treated by free and immobilized bacteria and the crude oil blank samples are shown in Fig 8. Fig 9 shows the comparison of normal alkanes content of the three samples. From these graphs, we can see that the free and the immobilized bacterial consortium behaved differently in degradation of normal alkanes. The content of *n*-C12 in crude oil samples treated by free bacterial consortium was higher than that of the blank in crude oil. Possibly, the free bacteria in the crude oil degradation transformed the long chain alkyls into *n*-C12, thereby resulting in *n*-C12 accumulation. The degradation efficiencies of normal alkanes of free bacterial consortium were 53.5%. Immobilization positively affects the degradation of the major normal alkanes. After immobilization, the degradation efficiency of normal alkanes was 85.4%, which increased by 31.9% comparing with the free cells. The effect of immobilization on the efficiency of the free bacterial consortium was significant. This observation may be caused by strong adsorption of immobilized carrier, leading to bio-utilization efficiently of normal alkanes.

**Fig 10:**
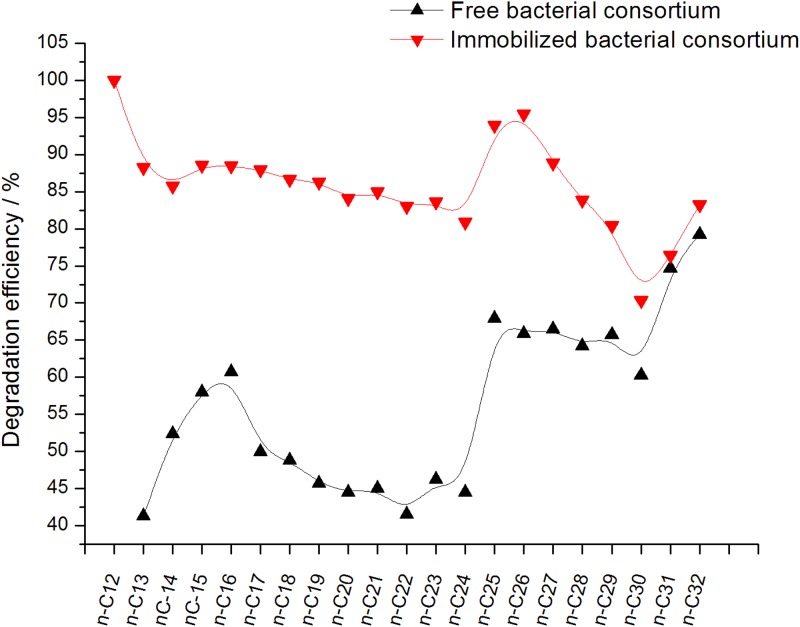
Degradation efficiency of the main normal alkanes by the free bacteria group and the immobilized bacteria group.

The degradation efficiency of normal alkanes by free and immobilized bacterial consortium was shown in Fig 10. The degradation of normal alkanes by free bacterial consortium was divided into four groups. The degradation efficiency of *n*-C13 to *n*-C16 gradually increased from 41.3% to 60.7%. The degradation efficiency of *n*-C17 to *n*-C24 remained at about 44%. The degradation efficiency of *n*-C25 to *n*-C30 was higher, with values of 60.2% to 67.9%. The degradation efficiency of *n*-C30 to *n*-C32 gradually increased, with values of 60.2% to 79.2%. The degradation effects of the immobilized bacterial consortium on normal alkanes were as follows. From *n*-C12 to *n*-C14, the degradation efficiency decreased from 100% to 85.6% and then stabilized from *n*-C14 to *n*-C24. Afterward, degradation efficiency increased gradually from *n*-C24 to *n*-C26, and then from *n*-C27 to *n*-C30, it was gradually reduced for a while before increasing again from *n*-C30 to *n*-C32.

#### PAHs

**Fig 11:**
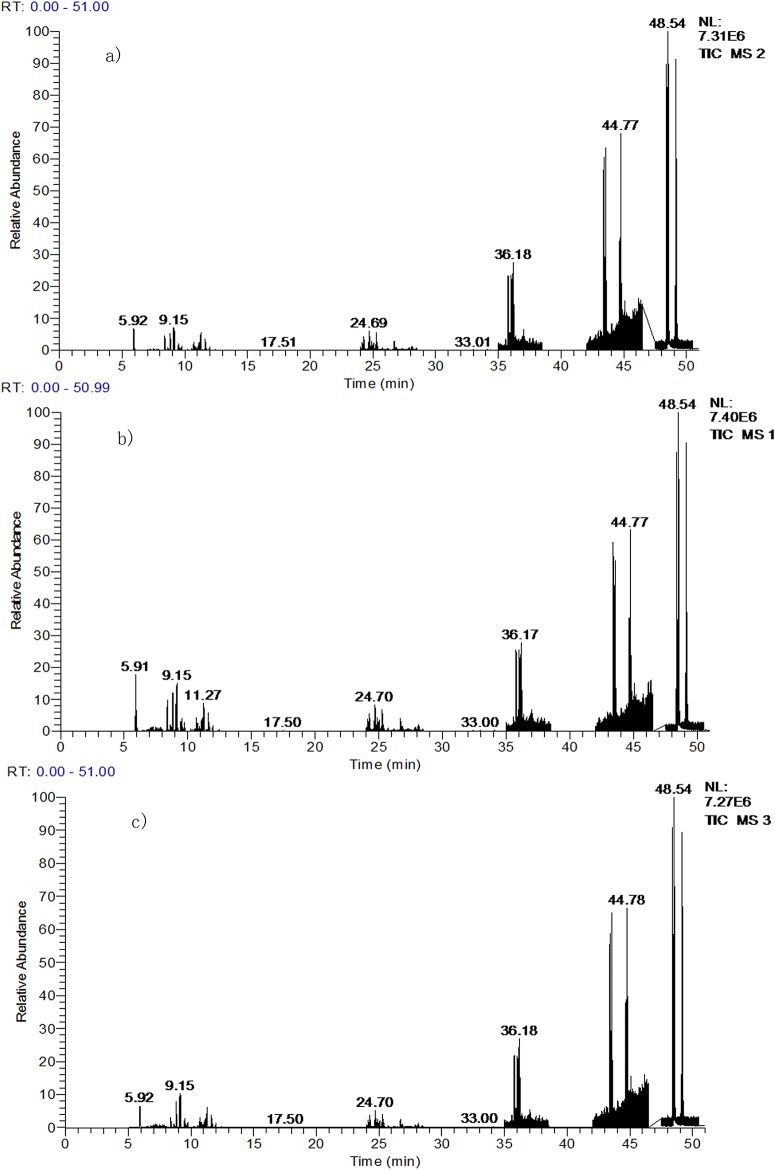
PAHs gas chromatograms in crude oil samples before and after biodegradation. a) the crude oil control; b) degradation sample of free bacterial consortium; c) degradation sample of immobilized bacterial consortium.

**Fig 12:**
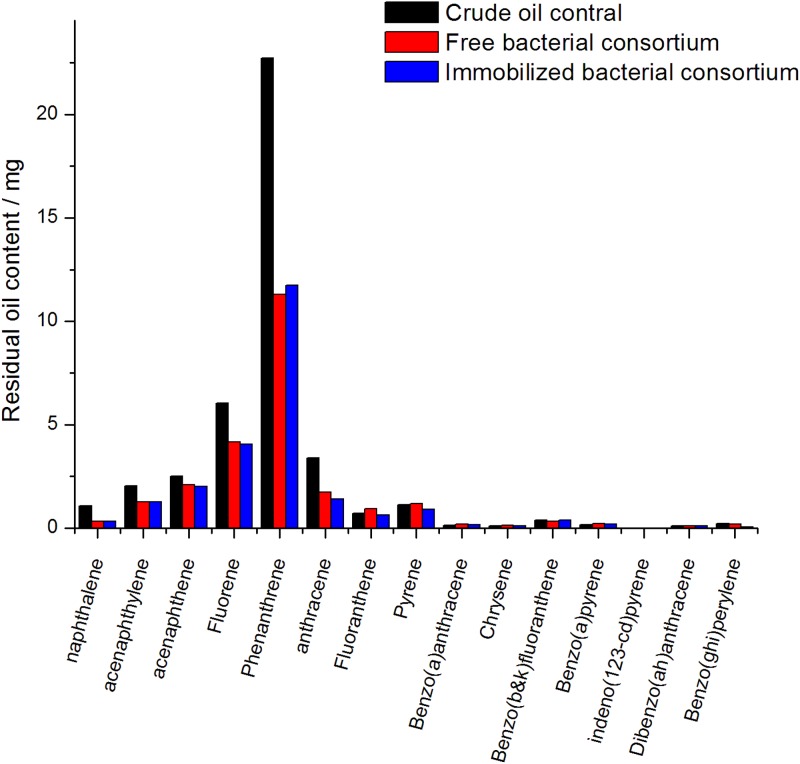
Degradation of the major PAHs by free bacteria and immobilized bacteria.

The gas chromatograms of PAHs of biodegradation by the free and immobilized cells are shown in Fig 11. As seen in Fig 12, the content of 16 kinds of common PAHs is very low in the crude oil blank samples. Among them, nine kinds (naphthalene, acenaphthylene, acenaphthene, fluorene, phenanthrene, anthracene, fluoranthene, pyrene, and benzo fluoranthene(b)) showed higher amount. phenanthrene was the highest among the nine PAHs. For simple structure of polycyclic aromatic hydrocarbons, such as naphthalene, phenanthrene, anthracene, the bacterial consortium showed positive degradation ability, with the degradation efficiencies above 50%. While the complex structure PAHs, such as fluoranthene, pyrene chrysene and benzo(b&k)fluoranthene showed low or even negative biodegradation efficiency. Previous studies showed the similar results in the work of Pasumarthi et al. (2013) [26] and Li et al. (2016). [28]

Immobilization improved the biodegradability of anthracene, fluoranthene and pyrene, the degradation efficiency increased by 9.1%, 40.0% and 24.2% respectively. Meanwhile, more complex structure PAHs, such as benzo(a)anthracene, chrysenes, benzo(b)fluoranthene, benzo(g,h,i)perylene and dibenzo(a,h)anthracene remained non-biodegraded, which may be due to their low aqueous solubility. [26, 29] The degradation efficiency of 16 typical PAHs by the free and immobilized bacteria were 40.6% and 42.5%, respectively. After immobilization, the biodegradation efficiency of PAHs increased by 1.9%. The effect of immobilization on the PAHs biodegradation efficiency of the free bacterial consortium was less significant than that of normal alkanes.

## Discussion

The microorganisms with efficient crude oil biodegradability, which were belonging to *Exiguobacterium* sp., *Alcaligenes* sp. *Bacillus* sp., or *Pseudomonas aeruginosa*, have been already reported in petroleum hydrocarbons biodegradation in marine enviroment. [26, 28, 30, 31] Biosurfactant generated from *Exiguobacterium*, *Bacillus*, *and Pseudomonas aeruginosa* were also found in previous study, [32, 33] which could enhance oil biodegradation efficiency. [21] The bacteria belonging to *Exiguobacterium* sp. and *Bacillus* sp. show strong biodegradation ability and have been reported in degrading chlorinated aromatic compounds that resistant to be biodegraded. [34–36] And in this study, these five strains belonging to bacterial species mentioned above all performed well in crude oil biodegradation, which got more than 50% of degradation efficiencies with 1% (*w/v*) crude oil. Flora can form a relative complete system for biodegradation. Through this way, a variety of enzymatic activities can aggregate for the expansion of the range of biodegradable petroleum fractions, and thus, the degradation activity can be improved. Bacterial consortia were formed up based on combinations of the five bacteria. However, not all the combinations showed cooperation actions in biodegradation, and the degradation efficiencies were lower than the single ones. The similar phenomenon was in previous study by Li et al. (2016). [28] That due to the competition between bacteria in a consortium result in the reduce of biodegradation efficiency. [37] The combinations of five strains performed the best cooperation actions, which got 75.1% of degradation efficiencies with 1% (*w/v*) crude oil. Crude oil degradation consortium has been carried out in previous studies. The comments cannot be carried out directly on the biodegradation ability of bacteria, due to various incubation conditions. In Varjani's study, about 30% crude oil (3% v/v) biodegradation occurred in 12 d. [38] There is 51.9% crude oil (2% *w/v*) degraded after 7d of incubation in Li’s work. [28] However, the selected bacterial consortium in the present study performed effectively compared with previous studies. About 68% of biodegradation efficiency of crude oil (2% *w/v*) obtained after 1 week of cultivation.

In the present study, free cells showed poor performance in lower incubated temperature (<15°C) and acid conditions (pH<7.0), [18] however, better performance of free cells occurred in the salinity various from 15 to 35‰. That may be concerned with their original growth environment. The five bacteria were from sea area adjacent to the Yangtze estuary, the Yangtze diluted water affected the salinity of seawater, which made the salinity various from 18 to 28‰. [39] And these strains adapted to the variations of seawater salinity. Immobilized bacterial consortium performed better than free bacteria in variations of environmental factors containing incubated temperature, initial pH and salinity of the medium. This agreed with previous work by Lin et al., 2014. [18] After 7d’s biodegradation, the degradation efficiencies of crude oil (2%, *w/v*) of immobilized cells were more than 60% with temperature of 15–30°C, initial pH of 6.0–9.0, salinity of 15%–35%. Immobilization improved microbial tolerance to high concentrations of harmful substances, [13] more obviously occurred when crude oil concentration more than 10% (*w/v*). The degradation of immobilized bacteria went stabilization phase with less degradation time (5 d), compared with that of the free ones (7d) and also increase the efficiency significantly.

Through analysis of normal alkanes biodegradation, the results suggested that the degradation efficiency of *n*-C17 to *n*-C24 in the crude oil samples was less than that of mostly short chains (<*n*-C17) and long chains (>*n*-C24). The possible causes may be as follows. The microorganism can use the short chains (<*n*-C17) at a faster rate than middle chains (*n*-C17 to *n*-C24), and biodegradation of higher molecular weight hydrocarbon fractions (>*n*-C24) to the middle chains leading to *n*-C17 to *n*-C24 accumulation. [28] Such result can be associated with the effect of free bacteria on the degradation of crude oil for 7 d. Different degrees of retention of intermediate products were observed. With longer duration, the free microbes can further degrade the normal alkanes. This is different from the patterns observed in Varjani's study, the biodegradation efficiency of C19–C28 is higher than C13–C18, [38] and similar phenomenon obtained in Li’s study. [28]

For free cells, the degradation efficiency of normal alkanes (53.5%) was more than that of 16 PAHs (40.6%) in present study. Improvement in the removal efficiency of normal alkanes (31.9%) by immobilization was also apparently high than that of PAHs (1.9%). That may be the shot time of biodegradation with only 7d’s incubation. Due to their structural complexity, biotransformation and biodegradation of aromatic hydrocarbons requires longer periods of time to occur. [29, 40] Previous biodegradation studies were taken in 30d [29], 45d [26] or even several months, [41] and better results have been obtained.

## Conclusion

In this study, five oil-degrading bacterial strains (ASW-1, ASW-2, ASW-3, ASS-1 and ASS-2) were isolated from oil polluted seawater samples nearby Zhejiang coast in China. They were affiliated to *Exiguobacterium* sp., *Pseudomonas aeruginosa*, *Alcaligenes* sp., *Alcaligenes* sp., and *Bacillus* sp., respectively. The mixed flora of the five strains showed the highest degradation efficiency of petroleum hydrocarbons of 75.1%.

Immobilized bacterial consortium performed better than free bacteria in variations of environmental factors containing incubated temperature, initial pH and salinity of the medium. After 7d’s biodegradation, the degradation efficiencies of crude oil (2%, *w/v*) of immobilized cells were more than 60% with temperature of 15–30°C, initial pH of 6.0–9.0, salinity of 15%–35%. Immobilization improved microbial tolerance to high crude oil concentrations, more obviously occurred when crude oil concentration more than 10% (*w/v*). The degradation process of crude oil by immobilized bacteria was accelerated compared with that of the free bacteria. SEM photos showed that activated carbons adding in the bio-carrier of sodium alginate-calcium chloride profited the diffusion of oxygen, substrates and metabolites, which led to better performance in biodegradation. Biodegradation of bacterial consortium in bio-carrier was the main moving force to crude oil removal, and the bio-carrier provided an arena for bacterial cells protection and better biodegradation.

The degradation efficiency of *n*-alkanes (53.5%) by free bacterial consortium was more than that of 16 PAHs (40.6%). Immobilized microorganisms performed better on biodegradation of normal alkanes than that of PAHs. Improvement in the removal efficiency of *n*-alkanes (31.9%) by immobilization was also apparently high than that of PAHs (1.9%).

## Supporting information

S1 File Partial 16S ribosomal DNA sequences of ASW-1. (TXT)

S2 File Partial 16S ribosomal DNA sequences of ASW-2. (TXT)

S3 File Partial 16S ribosomal DNA sequences of ASW-3. (TXT)

S4 File Partial 16S ribosomal DNA sequences of ASS-1. (TXT)

S5 File Partial 16S ribosomal DNA sequences of ASS-2. (TXT)
